# Association between microbial characteristics and poor outcomes among patients with methicillin-resistant *Staphylococcus aureus* pneumonia: a retrospective cohort study

**DOI:** 10.1186/s13756-015-0092-1

**Published:** 2015-12-14

**Authors:** Jennifer S. McDanel, Eli N. Perencevich, Daniel J. Diekema, Patricia L. Winokur, J. Kristie Johnson, Loreen A. Herwaldt, Tara C. Smith, Elizabeth A. Chrischilles, Jeffrey D. Dawson, Marin L. Schweizer

**Affiliations:** Department of Epidemiology, College of Public Health, University of Iowa, Iowa City, IA USA; Department of Internal Medicine, Carver College of Medicine, University of Iowa, Iowa City, IA USA; Iowa City Veterans Affairs Health Care System, Iowa City, IA USA; Department of Pathology, Carver College of Medicine, University of Iowa, Iowa City, IA USA; Clinical Quality, Safety, and Performance Improvement, University of Iowa Hospitals and Clinics, Iowa City, IA USA; Department of Pathology, University of Maryland School of Medicine, Baltimore, MD USA; Department of Biostatistics, College of Public Health, University of Iowa, Iowa City, IA USA; Present address: Department of Biostatistics, Environmental Health Sciences, and Epidemiology, Kent State University, Kent, OH USA

**Keywords:** MRSA pneumonia, Panton-Valentine leukocidin, Accessory Gene Regulator, mortality, length of stay

## Abstract

**Background:**

Methicillin-resistant *S. aureus* (MRSA) pneumonia is associated with poor clinical outcomes. This study examined the association between microbial characteristics and poor outcomes among patients with methicillin-resistant *Staphylococcus aureus* pneumonia.

**Findings:**

This retrospective cohort study included 75 patients with MRSA pneumonia who were admitted to two large tertiary care medical centers during 2003–2010. Multivariable models were created using Cox proportional hazards regression and ordinal logistic regression to identify predictors of mortality or increased length of stay (LOS). None of the microbial characteristics (PFGE type, *agr* dysfunction, SCC*mec* type, and detection of PVL, ACME, and TSST-1) were significantly associated with 30-day mortality or post-infection hospital length of stay, after adjusting for gender, age, previous hospital admission within 12 months, previous MRSA infection or colonization, positive influenza test, Charlson Comorbidity Index score, and treatment (linezolid or vancomycin).

**Conclusion:**

Large prospective studies are needed to examine the impact of microbial characteristics on the risk of death and other adverse outcomes among patients with MRSA pneumonia.

**Electronic supplementary material:**

The online version of this article (doi:10.1186/s13756-015-0092-1) contains supplementary material, which is available to authorized users.

## Findings

The literature is inconsistent regarding the association between microbial characteristics and poor outcomes among patients with methicillin-resistant *Staphylococcus aureus* (MRSA) pneumonia. Prior studies have linked the Panton-Valentine leukocidin (PVL) toxin and accessory gene regulator (*agr*) locus with necrosis of the lung [1, 2]. Additionally, some antimicrobials, such as linezolid or clindamycin, used to treat MRSA pneumonia may inhibit specific microbial virulence mechanisms [3, 4]. This study evaluated a cohort of patients with MRSA pneumonia to assess the association between MRSA microbial characteristics and poor outcomes such as death or increased hospital length of stay (LOS).

This retrospective study included patients admitted to the University of Iowa Hospitals and Clinics (UIHC) or to the University of Maryland Medical Center (UMMC) during 2003–2010. Patients were initially identified by *International Classification of Diseases, 9*^*th*^*Revision, Clinical Modification* (*ICD-9-CM*) codes for influenza-like illness [5] and were included if they had an ICD-9-CM code for pneumonia (ICD-9-CM codes: 480–488, V12.61, 997.31), had a banked MRSA isolate from either a respiratory or a blood culture during their admissions, and began antibiotic therapy with vancomycin or linezolid within 2 days before until fourteen days after the collection of the first MRSA positive culture. This study was approved by the institutional review board of the University of Iowa.

Antimicrobial susceptibility testing was performed using broth dilution as described by the Clinical and Laboratory Standards Institute [6]. Isolates were tested for susceptibility to tetracycline, trimethoprim-sulfamethoxazole, tigecycline, levofloxacin, linezolid, daptomycin, and vancomycin. Heterogeneous vancomycin-intermediate *S. aureus* (hVISA) screen testing was performed according to previously published methods [7]. The methods for pulsed-field gel electrophoresis (PFGE) are previously described, and a similarity coefficient of 80 % determined genetic relatedness [8, 9]. The *agr* screening test was performed by measuring the expression of δ-hemolysin using a β-lysin disk [10]. Polymerase chain reaction testing was used to identify the Panton-Valentine leukocidin (PVL) gene [9, 11], toxic shock syndrome toxin-1 (TSST-1) gene [9, 12], arginine catabolic mobile element (ACME) [9, 13], and staphylococcal cassette chromosome *mec* (SCC*mec*) [9] types I to V.

The primary outcomes analyzed were 30-day all-cause in-hospital mortality and post-infection hospital LOS. Thirty-day mortality was defined as death occurring within 30 days after the collection of the first MRSA positive respiratory or blood culture. Hospital LOS was measured beginning on the day the first MRSA positive respiratory or blood culture was collected until either hospital discharge or death. Comorbidities were measured using the Charlson Comorbidity Index [14]. Age was dichotomized on the median. Information was collected on hospital admission within the previous 12 months, previous MRSA infection or colonization, and having a positive influenza test during hospital admission. Hospital-acquired infections were defined as the first MRSA positive respiratory or blood culture collected more than 2 days after hospital admission.

Bivariable analyses were conducted using either Student’s t-test or the Wilcoxon Rank Sum test for continuous variables and either the chi-square test or Fisher’s exact test for categorical variables. Cox proportional hazard regression and ordinal logistic regression were used to perform the multivariable analyses assessing the association between microbial factors and 30-day mortality or LOS. Patients who did not die were categorized based on their length of stay: 0–3 days, 4–10 days, 11–20 days, and ≥21 days. Deceased patients were placed in a separate category since they had the worst outcome and varying lengths of stay before death. Variables with *P*<0.25 in the bivariable analysis were entered into the model using a manual stepwise method, and remained in the multivariable model if they were statistically significant (*P*<0.05). Data were analyzed using SAS software (SAS Institute, Cary, NC) version 9.3.

The cohort was comprised of 75 patients with MRSA pneumonia. The majority of patients were male (61 %) and the median age was 54. Twenty-four percent (18/75) of the patients died and the median post-infection LOS in the hospital was 9 days (interquartile range: 3–20). Most isolates were from respiratory cultures, including bronchial washes (9 %), tracheal aspirates (7 %), and sputum cultures (83 %).

Patients who survived were more likely than patients who died to be infected with MRSA isolates that were PVL-positive (42 % vs. 28 %; *P* = 0.277) or ACME-positive (39 % vs. 17 %; *P* = 0.085). Patients who died were more likely than those who survived to be infected with MRSA isolates with a dysfunctional *agr* (22 % vs. 16 %; *P* = 0.530). However, none of these results reached statistical significance (Table 1). Most MRSA isolates were susceptible to all antimicrobials tested except levofloxacin (9 % susceptible). All isolates were susceptible to vancomycin [minimum inhibitory concentration (MIC) range: 0.5–1 μg/mL] and linezolid (MIC range: 0.5–2 μg/mL) [Additional file: Susceptibility to antimicrobial agents of MRSA isolates from patients with MRSA pneumonia (*N* = 75) (see Additional file 1)]. None of the microbial characteristics were statistically significantly associated with increased post-infection LOS or mortality in the multivariable analyses statistically adjusting for gender, age, previous hospital admission within 12 months, previous MRSA infection or colonization, positive influenza test, Charlson Comorbidity Index score, and treatment (linezolid or vancomycin) (Table 2).

**Table 1:** Unadjusted associations for mortality or post-infection length of stay among patients with MRSA pneumonia

	Proportion of patients with characteristic		30-Day-In-Hospital mortality^a^		Post-infection length of stay^b^	
Characteristics	Patients who died *N* = 18	Patients who survived *N* = 57	Hazards ratio (95 % Confidence Interval)	*P*-value	Odds ratio (95 % Confidence Interval)	*P*-value
Patient characteristics						
Gender: Female	9 (50 %)	20 (35 %)	1.81 (0.72–4.57)	0.201	1.44 (0.63–3.30)	0.385
Age ≥55 years			4.69 (1.36–16.22)	0.015	2.15 (0.95–4.87)	0.068
Hospital admission within previous 12 months	6 (33 %)	31 (54 %)	0.52 (0.19–1.37)	0.185	0.40 (0.17–0.91)	0.028
Previous MRSA infection or colonization	6 (33 %)	11 (19 %)	1.82 (0.68–4.88)	0.234	1.31 (0.50–3.42)	0.583
Positive influenza test	2 (11 %)	3 (5 %)	1.69 (0.39–7.38)	0.488	2.13 (0.41–11.01)	0.368
Charlson comorbidity index score- median (IQR)	2 (1–3)	2 (1–4)	0.99 (0.83–1.20)	0.948	0.99 (0.84–1.16)	0.896
Hospital-acquired infection	6 (33 %)	19 (33 %)	0.85 (0.32–2.29)	0.752	1.50 (0.64–3.53)	0.354
Antimicrobial prescribed						
Linezolid	2 (11 %)	12 (21 %)	0.60 (0.14–2.60)	0.492	0.36 (0.13–1.03)	0.056
Vancomycin	17 (94 %)	54 (95 %)	0.61 (0.08–4.68)	0.223	3.10 (0.51–19.02)	0.221
Both linezolid and vancomycin	1 (6 %)	9 (16 %)	0.37 (0.05–2.76)	0.331	0.43 (0.13–1.41)	0.162
Microbial characteristics						
Panton-Valentine leukocidin	5 (28 %)	24 (42 %)	0.82 (0.36–1.88)	0.645	0.82 (0.36–1.88)	0.645
SCC*mec*						
Type II	12 (67 %)	27 (47 %)	1.22 (0.54–2.72)	0.634	1.22 (0.54–2.72)	0.634
Type IV	6 (33 %)	29 (51 %)	0.79 (0.35–1.76)	0.555	0.79 (0.35–1.76)	0.555
*agr* dysfunction	4 (22 %)	9 (16 %)	1.35 (0.44–4.10)	0.598	1.59 (0.54–4.62)	0.399
Arginine catabolic mobile element	3 (17 %)	22 (39 %)	0.35 (0.10–1.20)	0.094	0.70 (0.30–1.64)	0.413
Toxic shock syndrome toxin-1	1 (6 %)	2 (4 %)	1.22 (0.16–9.19)	0.846	2.35 (0.29–19.27)	0.423
PFGE type						
USA100	11 (61 %)	25 (44 %)	1.87 (0.72–4.82)	0.197	1.20 (0.54–2.69)	0.652
USA300	4 (22 %)	25 (44 %)	0.40 (0.13–1.21)	0.104	0.66 (0.29–1.51)	0.323
Other	3 (17 %)	7 (12 %)	1.40 (0.41–4.85)	0.594	1.57 (0.48–5.16)	0.458

*IQR* interquartile range, *SCCmec* staphylococcal cassette chromosome *mec*, *agr* accessory gene regulator, *PFGE* pulsed-field gel electrophoresis, *hVISA* heterogeneous vancomycin-intermediate *S. aureus*

^a^Defined as death occurring within the first 30 days after the day when the first MRSA positive respiratory or blood culture was collected

^b^Defined as the day the first MRSA positive respiratory or blood culture was collected until the day the patient was either discharged from the hospital or died

**Table 2:** Adjusted associations for mortality or post-infection length of stay among patients with MRSA pneumonia^a^

	30-Day-In-Hospital mortality^b^		Post-infection length of stay^c^	
Microbial characteristics	Hazards ratio (95 % Confidence Interval)	*P*-value	Odds ratio (95 % Confidence Interval)	*P*-value
Panton-Valentine leukocidin	0.72 (0.23–2.25)	0.566	0.67 (0.27–1.69)	0.398
SCC*mec*				
Type II	1.66 (0.53–5.16)	0.383	1.77 (0.69–4.52)	0.235
Type IV	0.62 (0.20–1.93)	0.414	0.51 (0.20–1.30)	0.158
*agr* dysfunction	1.84 (0.54–6.20)	0.323	1.30 (0.42–4.05)	0.648
Arginine catabolic mobile element	0.37 (0.10–1.47)	0.158	0.57 (0.22–1.48)	0.250
Toxic shock syndrome toxin-1	1.24 (0.14–11.10)	0.847	2.86 (0.26–31.18)	0.389
PFGE type				
USA100	1.29 (0.42–3.95)	0.656	1.70 (0.67–4.33)	0.268
USA300	0.54 (0.15–1.92)	0.344	0.60 (0.24–1.50)	0.269

*IQR* interquartile range, *SCCmec* staphylococcal cassette chromosome *mec*, *agr* accessory gene regulator, *PFGE* pulsed-field gel electrophoresis

^a^Adjusted for gender, age, previous hospital admission within 12 months, previous MRSA infection or colonization, positive influenza test, Charlson Comorbidity Index score, and treatment (linezolid or vancomycin)

^b^Defined as death occurring within the first 30 days after the day when the first MRSA positive respiratory or blood culture was collected

^c^ Defined as the day the first MRSA positive respiratory or blood culture was collected until the day the patient was either discharged from the hospital or died

Sixty-five patients (87 %) were infected with either a USA300 strain or a USA100 strain. Patients infected with a USA300 strain were less likely to receive linezolid (7 % vs. 31 %; *P* = 0.018) and were more likely to be younger (<55 years) [38 % vs. 67 %; *P* = 0.021] compared with patients infected with a USA100 strain (Table 3). Additionally, 14 % of the patients infected with a USA300 strain died compared with 31 % of the patients infected with a USA100 strain (*P* = 0.111).

**Table 3:** Patient characteristics, microbial characteristics, or treatments associated with strain type (*N* = 65)

Characteristics	USA100 *N* = 36	USA300 *N* = 29	*P*-value
Patient characteristics			
Gender: Female	16 (44 %)	9 (31 %)	0.269
Age ≥55 years	24 (67 %)	11 (38 %)	0.021
Hospital admission within previous 12 months	21 (58 %)	11 (38 %)	0.102
Hospital-acquired infection	10 (28 %)	12 (41 %)	0.249
Previous MRSA infection or colonization	8 (22 %)	5 (17 %)	0.618
Positive influenza test	1 (3 %)	2 (7 %)	0.582
Post-infection length of stay: median (IQR), in days^a^	6 (2–18)	12 (4–24)	0.232
Charlson Comorbidity Index score: median (IQR)	2 (1–3)	2 (0–4)	0.778
30-day mortality^b^	11 (31 %)	4 (14 %)	0.111
Antimicrobial prescribed			
Linezolid	11 (31 %)	2 (7 %)	0.018
Vancomycin	33 (92 %)	29 (100 %)	0.247
Microbial characteristics			
*agr* dysfunction	8 (22 %)	4 (14 %)	0.384
Toxic shock syndrome toxin-1	1 (3 %)	0 (0 %)	1.000
hVISA	1 (3 %)	0 (0 %)	1.000
Arginine catabolic mobile element	1 (3 %)	23 (79 %)	<0.001
Panton-Valentine leukocidin	0 (0 %)	26 (90 %)	<0.001
SCC*mec*			
Type II	35 (97 %)	4 (14 %)	<0.001
Type IV	0 (0 %)	25 (86 %)	<0.001

*IQR* interquartile range, *SCCmec* staphylococcal cassette chromosome *mec*, *agr* accessory gene regulator, *PFGE* pulsed-field gel electrophoresis, *hVISA* heterogeneous vancomycin-intermediate *S. aureus*

^a^Defined as the day the first respiratory or blood culture that grew MRSA was collected until the day the patient was either discharged from the hospital or died

^b^Defined as death occurring within the first 30 days after the day when the first respiratory or blood culture that grew MRSA was obtained

Numerous investigators have tried to determine whether specific microbial characteristics of MRSA infecting isolates are associated with poor outcomes [1, 2, 15, 16]. However, the results of those studies vary substantially. Even though this study was larger than most studies to examine microbial characteristics associated with MRSA pneumonia, this study did not identify any statistically significant associations between the microbial characteristics and outcomes. Even though the risk estimates suggested an association between a few of the microbial characteristics and death or increased post-infection LOS, the confidence intervals were wide, potentially due to the limited sample size and small number of patients who died. However, among patients infected with USA100 strains or USA300 strains, there was a trend toward increased mortality among those infected with USA100 strains.

Our study had limitations. First, patients might have been colonized in the throat or upper respiratory tract rather than having MRSA respiratory infections. To identify MRSA infected patients, this study only included patients who received vancomycin or linezolid (agents active against MRSA). Second, influenza-like illness ICD-9-CM codes were used to identify the study population because the patients initially were included in a study of influenza-like illness and MRSA pneumonia, thus this cohort may be subject to selection bias. However, this is not a large concern since cough was the most common influenza-like illness code identified. Finally, since this is a retrospective study, patients who had pneumonia but did not have a culture collected during their admission would not be included in the study. Large prospective studies are needed to assess whether microbial factors in combination with treatment factors or patient factors increase the risk of death and other adverse outcomes.

## Additional file

Additional file 1.
**Susceptibility to antimicrobial agents of MRSA isolates from patients with MRSA pneumonia (** ***N*** **= 75).** The susceptibility results for the 75 isolates that were tested to tetracycline, trimethoprim-sulfamethoxazole, tigecycline, levofloxacin, linezolid, daptomycin, and vancomycin. (DOCX 14 kb)
