# Supplementary material for: Association between microbial characteristics and poor outcomes among patients with methicillin-resistant Staphylococcus aureus pneumonia: a retrospective cohort study
Source: Antimicrob Resist Infect Control. 2015 Dec 14;4:51. doi: 10.1186/s13756-015-0092-1 (PMC4677450; doi:10.1186/s13756-015-0092-1)
Supplement: Additional file 1. — Susceptibility to antimicrobial agents of MRSA isolates from patients with MRSA pneumonia ( N = 75). The susceptibility results for the 75 isolates that were tested to tetracycline, trimethoprim-sulfamethoxazole, tigecycline, levofloxacin, linezolid, daptomycin, and vancomycin. (DOCX 14 kb) [file 13756_2015_92_MOESM1_ESM.docx]

Additional file 1: Susceptibility to antimicrobial agents of MRSA isolates from patients with MRSA pneumonia (N = 75)

| **Antimicrobial** | **MIC^a^ Range**  **(µg/mL)** | **MIC50**  **(µg/mL)** | **MIC90**  **(µg/mL)** | **% Resistant** |
| --- | --- | --- | --- | --- |
| Tetracycline | 0.12 - > 64 | 0.25 | 4 | 9 |
| Trimethoprim/Sulfamethoxazole | ≤ 0.03 - 32 | 0.06 | 0.06 | 4 |
| Tigecycline | 0.06 - 0.5 | 0.06 | 0.12 | N/A |
| Levofloxacin | 0.25 - > 64 | 32 | > 64 | 91 |
| Linezolid | 0.5 - 2 | 1 | 2 | 0 |
| Vancomycin | 0.5 - 1 | 1 | 1 | 0 |
| Daptomycin | 0.25 - 2 | 0.5 | 0.5 | N/A |

^a^ Minimum inhibitory concentration.
